# Biological Activities and Safety of *Citrus* spp. Essential Oils

**DOI:** 10.3390/ijms19071966

**Published:** 2018-07-05

**Authors:** Noura S. Dosoky, William N. Setzer

**Affiliations:** 1 Aromatic Plant Research Center, 230 N 1200 E, Suite 102, Lehi, UT 84043, USA; ndosoky@aromaticplant.org; 2 Department of Chemistry, University of Alabama in Huntsville, Huntsville, AL 35899, USA

**Keywords:** sweet orange, bitter orange, neroli, orange petitgrain, mandarin, lemon, lime, grapefruit, bergamot, yuzu, kumquat

## Abstract

*Citrus* fruits have been a commercially important crop for thousands of years. In addition, *Citrus* essential oils are valuable in the perfume, food, and beverage industries, and have also enjoyed use as aromatherapy and medicinal agents. This review summarizes the important biological activities and safety considerations of the essential oils of sweet orange (*Citrus sinensis*), bitter orange (*Citrus aurantium*), neroli (*Citrus aurantium*), orange petitgrain (*Citrus aurantium*), mandarin (*Citrus reticulata*), lemon (*Citrus limon*), lime (*Citrus aurantifolia*), grapefruit (*Citrus* × *paradisi*), bergamot (*Citrus bergamia*), Yuzu (*Citrus junos*), and kumquat (*Citrus japonica*).

## 1. Introduction

The genus *Citrus* (Rutaceae) is one of the ancient, most traded, and most popular crops. The earliest records of its cultivation date back to 2100 BC [1]. The origin of *Citrus* is still controversial; however, it is believed to have originated from Southeast Asia [2]. *Citrus* is grown widely all over the world for its numerous health benefits. *Citrus* fruits are consumed as a fresh fruit desert or used for making juice and jam. They are an excellent source of vitamins, especially vitamin C. Processing *Citrus* fruits results in a significant amount of waste (peels, seeds, and pulps), which accounts for 50% of the fruit [3]. *Citrus* waste is a valuable source of *d*-limonene, flavonoids, carotenoids, dietary fibers, soluble sugars, cellulose, hemicellulose, pectin, polyphenols, ascorbic acid, methane, and essential oils [4,5,6]. Interestingly, the essential oil (EO) is the most vital by-product of *Citrus* processing. *Citrus* EOs are broadly used as natural food additives in several food and beverage products [7] because they have been classified as generally recognized as safe (GRAS) [8]. Furthermore, *Citrus* EOs are used as natural preservatives due to their broad spectrum of biological activities including antimicrobial and antioxidant effects [9]. The presence of terpenes, flavonoids, carotenes, and coumarins is thought to be responsible for the strong anti-oxidative and antimicrobial activities [10,11,12,13,14]. Due to their pleasant refreshing smell and rich aroma, *Citrus* EOs are also used in air-fresheners, household cleaning products, perfumes, cosmetics, and medicines.

**Figure 1:**
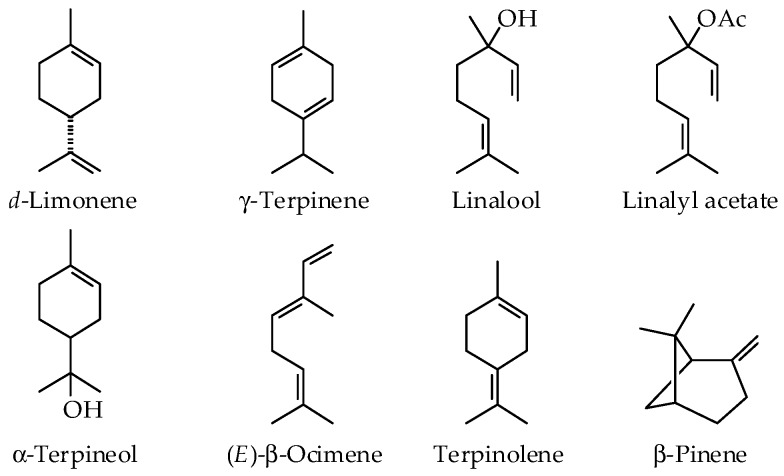
Chemical structures of key volatile components in *Citrus* essential oils.

**Figure 2:**
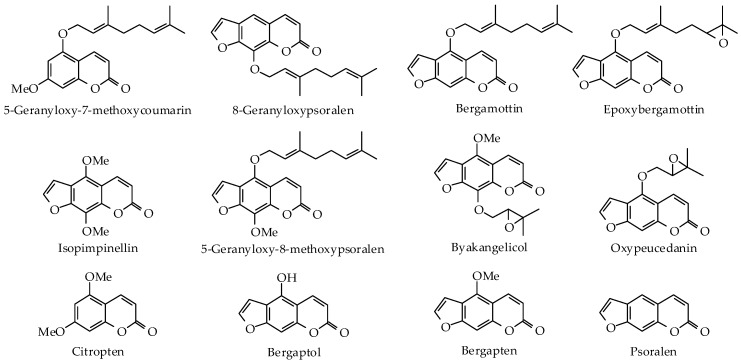
Chemical structures of key non-volatile components in expressed *Citrus* essential oils.

Because of their high economic importance, numerous studies have investigated the chemical composition of the peel, leaf, and flower essential oils of different *Citrus* species. It is worth noting that there is a great variation in the chemical composition of *Citrus* oils due to differences in origin, genetic background, season, climate, age, ripening stage, method of extraction, etc. [15,16,17,18,19]. The key volatile components are presented in Figure 1. Sweet orange, bitter orange, mandarin, and grapefruit EOs are rich in monoterpenes with the major component being *d*-limonene (65.3–95.9%) (Table 1) [8]. The main components in the essential oil of bitter orange leaf are linalyl acetate and linalool [16], while the flower EO contained linalool as the major component, followed by *d*-limonene and linalyl acetate [20]. Some of the *Citrus* EOs are prepared by expression, which results in the presence of non-volatile components (Figure 2) that can cause photosensitivity and skin irritation [8]. The percentages of these non-volatile constituents in expressed oils are given in Table 2.

The objective of this review is to summarize the reported biological activities and safety of the essential oils of sweet orange (*Citrus sinensis* L.), bitter orange (*Citrus aurantium* L.), neroli (*Citrus aurantium* L.), orange petitgrain (*Citrus aurantium* L.), mandarin (*Citrus reticulata* Blanco), lemon (*Citrus limon* Osbeck), lime (*Citrus aurantifolia*), grapefruit (*Citrus* × *paradisi* Macfady), bergamot (*Citrus bergamia* Risso & Poit), Yuzu (*Citrus junos* Sieb. ex Tanaka), and kumquat (*Citrus japonica* Thunb).

## 2. Biological Properties

A summary of the biological activities of different *Citrus* essential oils is presented in Table 3. 

### 2.1. Sweet Orange (Citrus sinensis L.) Essential Oil

Sweet orange EO showed anticarcinogenic potential via inducing apoptosis in human leukemia (HL-60) cells [28] and human colon cancer cells [29], and inhibiting angiogenesis and metastasis [29]. Olfactory stimulation using orange EO induced physiological and psychological relaxation. Inhalation of orange EO for 90 s caused a significant decrease in oxyhemoglobin concentration in the right prefrontal cortex of the brain which increases comfortable, relaxed, and natural feelings [30]. The odor of sweet orange decreases the symptoms of anxiety and improves the mood [31]. The oil showed strong anxiolytic activity in Wistar rats [32]. When female dental patients were exposed to sweet orange odor diffused in the waiting room prior to a dental procedure, they showed lower levels of state-anxiety compared to control patients who were exposed to air only [33]. Sweet orange EO in combination with ginger and accompanied by a massage was effective in alleviating moderate to severe knee pain among the elderly in Hong Kong [34]. Moreover, sweet orange EO suppressed pre-neoplastic hepatic lesions during *N*-nitrosodiethylamine (DEN)-induced hepatocarcinogenesis in rats by restoring the normal phenotype and upregulating junctional complexes [35]. Injections of orange EO in mice 24 h after subcutaneous injections with dibenzo-[α]-pyrene (DBP) reduced the tumor incidence to less than 50% after 30 weeks [36]. In addition, the oil was reported to have a good radical-scavenging activity [37], mainly due to the high *d*-limonene content [12,13]. It is used in combination with thyme oil to improve the quality traits of marinated chicken meat [38]. Moreover, formulations based on orange and sweet basil oils were effective in treating acne [39]. Improvement of the acne condition was observed with 43–75% clearance of lesions. It should be noted that there were some side effects, such as burning and redness that disappeared within a few minutes of completing the application [39]. Sweet orange EO was reported to inhibit the growth of several bacteria including *Staphylococcus aureus*, *Listeria monocytogenes*, *Vibrio parahaemolyticus*, *Salmonella typhimurium*, *Escherichia coli*, and *Pseudomonas aeruginosa* [40,41,42,43], as well as several fungal species, such as *Aspergillus flavus*, *A*. *fumigatus*, *A*. *niger*, *A*. *terreus*, *Alternaria alternata*, *Cladosporium herbarum*, *Curvularia lunata*, *Fusarium oxysporum*, *Helminthosporium oryzae*, *Penicillium chrysogenum*, *P*. *verrucosum*, and *Trichoderma viride* [10,44,45]. It also showed a good anti-aflatoxigenic effects (inhibited aflatoxin B_1_) at 500 ppm [44]. In addition, it has an intense larvicidal activity against the malaria vector, *Anopheles labranchiae* [46], and the vector of yellow and dengue fever, *Aedes aegypti* [47]. Sweet orange EO is a potent fumigant against house flies, cockroaches, and mosquitoes [48,49]. It can be used for controlling subterranean termites [50]. It is also an effective anthelmintic agent against gastrointestinal nematodes; five times more effective on *Haemonchus contortus* eggs than tea tree EO [51]. Moreover, sweet orange EO acted as a growth promoter, increased immunity, and improved disease resistance to *Streptococcus iniae* in Tilapia [52].

### 2.2. Bitter Orange (Citrus aurantium L.) Essential Oil

Bitter orange EO is used as a mild sedative and hypnotic for its soothing, calming, and motor relaxant effects [53]. It also enhances sleeping time and is used to treat insomnia [54]**.** Bitter orange odor decreases the symptoms of anxiety, improves mood, and creates a sense of well-being [53]. It showed strong anxiolytic activity in rodents without any motor impairment, even after 15 consecutive days of treatment [55]. It increased social interactions for rats (time spent in active social interaction), and increased exploration time in the open arms of the elevated plus-maze (EPM) [55]. It was also effective in treating the symptoms of anxiety in patients with chronic myeloid leukemia prior to the collection of medullary material [56]. It exerted its antianxiety effects by regulating serotonin (5-HT) receptors in rats [57] and its antidepressant effects through the monoaminergic system in mice [58]. Furthermore, bitter orange EO was effective in reducing the severity of first-stage labor pain and anxiety in primiparous women [59], as well as in alleviating moderate and severe knee pain [34]. Bitter orange EO is used as a natural antiseizure and anticonvulsant agent. It has been used in treating epilepsy and seizures [54]. It has been reported to have anti-spasmodic effect and to enhance sexual desire [59]. Due to the presence of limonene, bitter orange EO possesses its gastroprotective and ulcer healing actions through increasing the gastric production of mucus, which is useful as a secondary intervention in the treatment of chronic inflammatory diseases [60]. It is used as a treatment for digestive disorders such as slow digestion, constipation, flatulence, gastric problems, etc. [53]. Bitter orange EO suppressed preneoplastic hepatic lesions during DEN-induced hepatocarcinogenesis in rats by restoring the normal phenotype and upregulating junctional complexes [35]. Bitter orange EO showed good radical-scavenging activity [53], largely due to the high *d*-limonene content [12,13] and its microencapsulated form, which was effective in reducing oxidative stress in acute otitis media rats [61]. Due to its free radical-scavenging properties, bitter orange extract showed nephroprotective effects against gentamicin-induced renal damage [62]. The antibacterial activity of bitter orange EO was manifested by inhibiting the growth of *Listeria innocua*, *Salmonella enterica*, *Escherichia coli*, *Pseudomonas fluorescens*, and *Aeromonas hydrophila* [53,63,64]. It was also effective in controlling multi-species biofilms [65]. Due to its antimicrobial effects, bitter orange EO is used for treating colds, dull skin, flu, gums and mouth, and chronic bronchitis, as well as a food preservative [53]. The diluted oil is used to treat pimples and acne [53]. In addition, bitter orange EO inhibits the growth of *Penicillium digitatum*, and *P*. *italicum* [15,53]. The oil was mentioned as a topical treatment for skin fungal infections like ringworm, jock itch, and athlete’s foot [66]. Furthermore, bitter orange EO showed potent fumigant and anti-cholinesterase activities against the silverleaf whitefly, *Bemisia tabaci* [67]. It was also effective against the larvae of the malaria vector, *Anopheles labranchiae* [46].

### 2.3. Neroli (Citrus aurantium L.) Essential Oil

Neroli EO is used as a sedative for its soothing, calming, and motor relaxant effects by healthcare centers in Puerto Rico, Guatemala, Mexico, Italy, Martinique, and Spain [55]. Neroli EO is effective for cardiac palpitations resulting from shock or fear [68]. Similar to the fruit peel oil, the odor of neroli decreases the symptoms of anxiety, improves mood, and creates a sense of well-being [53]. It was proven to be effective in reducing preoperative anxiety before minor operations [69]. Neroli EO reduced the mean anxiety scores in postmenopausal women [70]. It exerted its antianxiety effects by regulating 5-HT receptors in rats [57]. Neroli EO is used as a natural antiseizure and anticonvulsant agent [71]. It has been used in treating insomnia, epilepsy, and seizures [72]. Neroli EO has central and peripheral antinociceptive effects which support the ethnomedicinal claims of its use in the management of pain and inflammation [73]. Neroli EO possesses significant anti-inflammatory activity against acute and chronic inflammation [73]. Neroli EO is effective in reducing stress and improving the endocrine system. Inhalation of neroli EO helps in relieving menopausal symptoms, reducing blood pressure, and increasing sexual desire in postmenopausal women [74]. It also decreased the overall symptoms of premenstrual syndrome (PMS) in university students. It showed positive effects on the mood, blood pressure, pain, inflammation, bloating, and indigestion in addition to its anti-depressant effects [75]. Inhaling neroli odor enhances sexual desire [59]. Neroli EO is an endothelium- and smooth-muscle-dependent vasodilator that can alleviate cardiovascular symptoms. The endothelial component is mediated by the nitric oxide to soluble guanylyl cyclase pathway, while the smooth muscle component involves inhibiting extracellular Ca^2+^ influx and store-operated Ca^2+^ release mediated by the ryanodine receptor (RyR) signaling pathway [76]. Inhaling a mixture of lavender, ylang-ylang, marjoram, and neroli (20:15:10:2) decreased systolic and diastolic blood pressure, as well as the concentration of salivary cortisol in prehypertensive and hypertensive subjects [77]. These positive effects were immediate and continuous [77]. Furthermore, neroli EO is a strong antioxidant. It showed a 100% singlet oxygen scavenging activity at all concentrations between 0.1 and 2% [20,78]. Interestingly, the *C*. *aurantium* flower extract showed anti-amnesic and repairing effects on memory, learning impairments, and behavioral disorders induced by scopolamine, and has the potential to treat Alzheimer’s disease [72]. Neroli EO inhibits the growth of several bacteria including *Bacillus subtilis*, *B*. *cereus*, *Staphylococcus aureus*, *S*. *epidermis*, *Enterococcus faecalis*, *Micrococcus luteus*, *Listeria monocytogenes*, *Salmonella enteritidis*, *Escherichia coli*, *Pseudomonas aeruginosa*, and *Klebsiella pneumonia* [53,79], as well as several fungi including *Aspergillus niger*, *A*. *flavus*, *A*. *nidulans*, *A*. *fumigatus*, *Fusarium graminearum*, *F*. *oxysporum*, *F. culmorum*, and *Alternaria alternata* [20,53,75,79].

### 2.4. Orange Petitgrain (Citrus aurantium L.) Essential Oil

Orange petitgrain EO showed a remarkable radical-scavenging activity, higher than the flower oil (neroli) and fruit peel oil (bitter orange) from the same plant [78,80]. The potent antioxidant effect could be attributed to the high *d*-limonene content [12,13]. It also inhibited the growth of *Bacillus subtilis*, *Staphylococcus aureus*, *Escherichia coli*, *Saccharomyces cerevisiae*, *Mucor ramannianus*, and *Fusarium culmorum* [81].

### 2.5. Mandarin (Citrus reticulata Blanco) Essential Oil

*Citrus reticulata* EO showed an anti-proliferative effect against human embryonic lung fibroblasts (HELFs) and showed protective effects against bleomycin (BLM)-induced pulmonary fibrosis in rats. The mechanism is thought to be through adjusting the unbalance of oxidation and antioxidation, down-regulating the expressions of connective tissue growth factor (CTGF) and mRNA in lung tissues, and reducing collagen deposition and fibrosis [82]. *C. reticulata* EO showed a moderate radical scavenging activity [83] mainly due to the high *d*-limonene content [12]. Mandarin oil is well known for its broad spectrum antibacterial and antifungal actions. It inhibits the growth of several bacteria including *Escherichia coli*, *Bacillus subtilis*, *Pseudomonas aeruginosa*, and *Staphylococcus aureus* [83,84], as well as several fungi including *Penicillium italicum*, *P*. *digitatum*, *P*. *chrysogenum*, *Aspergillus niger*, *A. flavus*, *Alternaria alternata*, *Rhizoctonia solani*, *Curvularia lunata*, *Fusarium oxysporum*, and *Helminthosporium oryzae* [84,85,86,87].

### 2.6. Lemon (Citrus limon Osbeck) Essential Oil

Lemon EO is a natural stress reliever. Inhaling lemon EO causes anti-stress effects through modulating the 5-HT and dopamine (DA) activities in mice [88,89]. Lemon EO showed cytotoxic effects against human prostate, lung, and breast cancer cells [90]. It also induced apoptosis in HL-60 cells due to the presence of citral, decanal, and octanal [28]. Oral administration of lemon EO inhibited 4-(methylnitrosamino)-1-(3-pyridyl)-1-butanone (NNK)-induced neoplasia of the lungs and forestomach of female mice [91]. Lemon EO causes activation of the sympathetic nerve activity innervating the white adipose tissue (WAT), which increases lipolysis and results in the suppression of body weight gain [92]. Lemon EO significantly reduces lipid peroxidation levels and nitrile content, but increases reduced glutathione (GSH) levels, as well as superoxide dismutase, catalase, and glutathione peroxidase activities in mouse hippocampus [93]. The neuroprotective effect of lemon EO is attributed to its remarkable radical-scavenging activity [94,95]. Prolonged exposure (for 2 weeks) to lemon EO induces significant changes in neuronal circuits involved in anxiety and pain in rats [96]. Lemon EO improves creativity and mood, and is thought to affect heart rhythm [97]. The analgesic effect of lemon EO is induced by dopamine-related activation of anterior cingulate cortex (ACC) and the descending pain inhibitory system [98]. Inhalation of lemon EO reduces the intensity of nausea and vomiting of pregnancy (NVP) by 33% [99]. It also showed anti-spasmodic activity [89]. Lemon EO significantly enhanced attention level, concentration, cognitive performance, mood, and memory of students during the learning process [100]. Rats exposed to lemon EO were able to find a target point faster than a control group [89]. Lemon EO is a safe and effective penetration enhancer for topical administration of lipid- and water-soluble vitamins which are critical issues for the protection of anti-ageing formulations. It significantly enhances the trans-epidermal release of α-tocopherol (vitamin E), retinyl acetate (vitamin A), pyridoxine (vitamin B_6_), and ascorbic acid (vitamin C) from topical emulsions in reconstructed human epidermis [101]. In addition, lemon EO is a potent antibacterial against *Bacillus cereus*, *Mycobacterium smegmatis*, *Listeria monocytogenes*, *Lactobacillus curvatus*, *L*. *sakei*, *Micrococcus luteus*, *Escherichia coli*, *Klebsiella pneumoniae*, *Pseudococcus aeruginosa*, *Proteus vulgaris*, *Enterobacter gergoviae*, *E*. *ammnigenus*, *Staphylococcus aureus*, *S*. *carnosus*, and *S*. *xylosus* [102,103], and a strong antifungal against *Aspergillus niger*, *A*. *flavus*, *Penicillium verrucosum*, *P*. *chrysogenum, Kluyveromyces fragilis*, *Rhodotorula rubra*, *Candida albicans*, *Hanseniaspora guilliermondii*, and *Debaryomyces hansenii* [10]. Lemon EO has insect repellent effects against the malaria vector, *Anopheles stephensi* [104]. It also showed remarkable miticidal activity against *Sarcoptes scabiei* var. *cuniculi*, both in vitro and in vivo. When lemon EO was tested at 20% and applied topically on the infected parts of rabbits once a week for four successive weeks, the infected rabbits completely recovered after the second week of treatment [105].

### 2.7. Key Lime (Citrus aurantifolia) Essential Oil

Lime EO has been used to relieve common cold, flu, asthma, arthritis, and bronchitis [111,164]. It could be useful in weight loss and the treatment of drug-induced obesity and related diseases. It displayed a reduction in body weight and food consumption in ketotifen-induced obese mice [106]. It has been reported as a potent spasmolytic agent [107,108]. Lime EO could also be useful in treating Alzheimer’s disease since it is a strong selective acetylcholinesterase and buytrylcholinesterase inhibitor [109]. It has a remarkable radical-scavenging activity (IC_50_ = 19.6 μg/mL) [109] due to the high *d*-limonene content [12,13]. Lime EO exhibited anti-inflammatory effects by reducing cell migration, cytokine production, and protein extravasation induced by carrageenan [110]. Lime EO is used as a flavoring agent in syrups and suspensions [111,112]. In addition, it is a potent antibacterial against *Escherichia coli*, *Listeria monocytogenes*, *Bacillus subtilis*, *Enterococcus durans*, *E*. *hirae*, *Staphylococcus epidermidis*, *S*. *aureus*, *Enterobacter cloacae*, *Pseudomonas aeruginosa*, *Serratia marcescens*, *Shigella flexnerii*, *Streptococcus faecalis*, *Citrobacter* spp., *Klebsiella pneumoniae*, *and Salmonella typhi* [111,113]. It also inhibits the growth of many fungi including *Colletotrichum gloeosporioides*, *Rhizopus stolonifer*, *Aspergillus niger*, *A*. *parasiticus*, *Rhizoctonia solani*, *Candida albicans*, and *C*. *parapsilosis* [111,113]. Lime EO has insecticidal activity (contact, fumigation, and feeding deterrent activities) against the maize weevil, *Sitophilus zeamais* [114]. It showed phytotoxic activities against *Avena fatua* L., *Echinochloa crus-galli* (L.) Beauv, *Allium cepa* L., and *Phalaris minor* Retz [113].

### 2.8. Grapefruit (Citrus × paradisi Macfady) Essential Oil

Because of its anti-obesity effects, grapefruit EO is called the “dieter’s friend” [116]. The fragrance of grapefruit EO causes activation of the sympathetic nerve activity innervating the WAT, which facilitates lipolysis, then results in a suppression of body weight gain [92,115]. It efficiently inhibits adipogenesis via inhibiting the accumulation of triglycerides [117]. When mixed with patchouli oil, grapefruit EO is known to lower cravings and hunger, which makes it a great tool to lose weight in a healthy way [116]. The bright, refreshing scent of grapefruit EO energizes and uplifts the senses. Grapefruit EO promotes body cleansing and removal of toxins and excess fluids [116]. Grapefruit EO was cytotoxic against human prostate and lung cancer cells [90]. It also induced apoptosis in HL-60 cells due to the presence of citral, decanal, and octanal [28]. Moreover, it showed a strong antibacterial activity against *Bacillus cereus*, *Enterococus faecalis*, *Escherichia coli*, *Klebsiella pneumoniae*, *Pseudococcus* sp., *Salmonella thyphimurium*, *Shigella flexneri*, and *Staphylococcus aureus* [118,119], and a strong antifungal activity against *Aspergillus niger*, *Candida albicans*, *Cladosporium cucumerinum*, *Penicillium digitatum*, *P*. *italicum*, and *P*. *chrysogenum* [118,119,120]. Grapefruit EO was 95% lethal to eggs and larvae of *Anastrepha fraterculus* and *Ceratitis capitata* [121]. It completely inhibited the viability of *Aedes aegypti* eggs exposed at 400 ppm, and inhibits its larval development at 100 ppm [122]. Also, grapefruit EO is a potent larvicide against *Anopheles stephensi* at 80 ppm [123]. It caused an 89.6% decrease of *Eimeria*-induced coccidiosis contamination with 5 mg/kg for 30 days [124].

### 2.9. Bergamot (Citrus bergamia Risso & Poit) Essential Oil

Bergamot EO is widely used in the perfumery, pharmaceutical, cosmetic, and food industries [125]. It is used in suntan preparations due to the presence of bergapten, which is the active melanogenic component [126]. Bergamot EO is used in complementary medicine to treat chronic nociceptive and neuropathic pain via modulating sensitive perception of pain [127,128,129]. Intraplantar injection of bergamot EO, linalool, and linalyl acetate showed a peripheral antinociception effect in the capsaicin test mediated by a peripheral opioid mechanism [129,130]. A combination of a low dose of morphine with inactive doses of bergamot oil or linalool was sufficient to induce antiallodynic effects in mice via inhibiting spinal extracellular signal-regulated protein kinase (ERK) phosphorylation [127,131]. The oil is used to facilitate wound healing [132]. Bergamot EO was reported to be cytotoxic against SH-SY5Y human neuroblastoma cells, suppressing their growth rate through a mechanism related to both apoptotic and necrotic cell death [133,134]. Bergamottin and 5-geranyloxy-7-methoxycoumarin were identified as the bioactive molecules responsible for the cytotoxic effect of bergamot EO [133]. Bergamot EO inhibited tumor formation by the carcinogen NDMA in vitro by more than 70% [136]. Bergamot oil and its *d*-Limonene were reported to modulate autophagic pathways in SH-SY5Y cells [125]. Liposomal bergamot oil showed improved anticancer activity against SH-SY5Y cells because of its higher stability and higher bioavailability [135]. In addition, it has been shown to reduce neuronal damage caused in vitro by excitotoxic stimuli by preventing an injury-induced decrease of phosphorylated protein kinase B (phospho-Akt) and phosphorylated glycogen synthase kinase 3β (phospho-GSK-3β) levels [137,138]. Bergamot EO is used as a mild sedative that acts by calming and soothing the nervous system [139]. In rodent experiments, the pleasant, refreshing odor of bergamot decreased the symptoms of stress-induced anxiety and minimized behavior-related depressive disorders in chronic stressed rats [139]. Inhalation of bergamot EO was reported to increase the release of amino acid neurotransmitters (glutamate, gamma-aminobutyric acid (GABA), aspartate, glycine, and taurine) in rat hippocampuses, both in vivo and in vitro, which suggested that the oil may interfere with exocytosis [165]. Similar to diazepam, bergamot oil exerted anxiolytic-like behaviors and attenuated hypothalamic-pituitary-adrenal (HPA) axis activity via reducing the corticosterone response to acute stress caused by EPM [140]. A pilot study performed in the waiting room of a mental health treatment center (Utah, USA) revealed that inhalation of bergamot EO for 15 minutes improves positive feelings [141]. Furthermore, bergamot EO showed a good radical scavenging activity evaluated by β-carotene bleaching test (IC_50_ = 42.6 µg/mL) [109] due to the high *d*-limonene content [12,13]. Bergamot EO inhibits the growth of several bacteria including *Escherichia coli*, *Staphylococcus aureus*, *Bacillus cereus*, *Salmonella enterica*, *S*. *typhimurium*, *Pseudomonas putida*, *Arcobacter butzleri*, *Enterococcus faecium*, *E*. *faecalis*, and *Listeria monocytogenes* [142,143,144]. Several studies showed a broad spectrum antifungal activity of bergamot EO against *Hanseniaspora guilliermondii*, *Debaryomyces hansenii*, *Kluyveromyces fragilis*, *Rhodotorula rubra*, *Candida albicans*, *Aspergillus niger*, *A*. *flavus*, *Penicillium italicum*, *Fusarium solani*, *F*. *sporotrichioides*, *F*. *oxysporum*, *Curvularia lunata*, *Verticillium dahliae*, *Phomopsis* sp., *Phoma* sp., and *Myrothechium verrucaria* [142,143,145]. It was also reported to have antifungal effects against dermatophytes of the genera *Trichophyton*, *Microsporum*, and *Epidermophyton* [146]. It could be used in the treatment of dermatophytosis in animals [147]. The mechanism underlying its antimicrobial and antifungal effect is thought to be via increasing reactive oxygen species (ROS) production, relevant to its action in human polymorphonuclear leukocytes [132]. Bergamot EO also showed strong antimycoplasmal activity against *Mycoplasma hominis*, *M*. *fermentans*, and *M*. *pneumoniae* [148].

### 2.10. Yuzu or Yuja (Citrus junos Sieb. ex Tanaka) Essential Oil

Yuzu EO inhibited the formation of the carcinogen *N*-nitrosodimethylamine (NDMA) in vegetables (by 22–59%) and saliva (by 24–62%) [149]. Yuzu EO is useful in treating bronchial asthma due to its anti-inflammatory activities. It inhibits the production of cytokines and ROS, and reduces eosinophil migration [150]. Yuzu odor was reported to decrease maternal anxiety for a sick child receiving an infusion at a pediatric clinic [151]. A 10 min inhalation of the yuzu odor significantly decreased the heart rate and increased the high frequency power of heart rate variability reflecting parasympathetic nervous system activity, regardless of menstrual phase. Inhalation of the yuzu oil decreased total mood disturbance, tension-anxiety, anger-hostility, and fatigue, which are common premenstrual symptoms [152,153]. Yuzu odor promotes mind and body health in Japan [152]. It is also used to suppress the odor of Niboshi soup stock [154]. Yuzu peel ethanol extract is useful in preventing colitis and colorectal cancer through reducing cyclooxygenase-2 (COX-2) expression [155]. This extract also showed hypocholesterolemic effect both in vitro and in vivo by reducing the weight gain, lipid accumulation, liver fat content, liver weight, total cholesterol, and low-density lipoprotein (LDL) cholesterol [156]. Yuzu extract was reported to exert anti-diabetic activity through increasing glucose uptake in C_2_C1_2_ myotubes by modulating the AMP-activated protein kinase (AMPK) and peroxisome proliferator-activated receptor gamma (PPAR-γ) signaling pathways. It improved insulin resistance (IR) in mice that were fed a high-fat diet [157]. Moreover, yuzu peel extract showed anti-obesity effects in a zebrafish model via activating hepatic PPAR-α and adipocyte PPAR-γ pathways [158]. The methanol extract of yuzu could be beneficial for individuals at high risk of cardiovascular disease because it inhibits platelet aggregation [159]. Yuzu extract could be useful in treating heart failure as it prevents myocardial infarction (MI)-induced ventricular dysfunction and structural remodeling of myocardium [160].

### 2.11. Kumquat (Citrus japonica Thunb) Essential Oil

Kumquat EO showed antiproliferative action against human prostate cancer (LNCaP) cells via inducing apoptosis and inhibition of inflammation [161]. The oil also showed a considerable radical-scavenging activity evaluated by a 2,2-diphenyl-1-picrylhydrazyl (DPPH) test [161,162] due to the high *d*-limonene content [12,13]. Kumquat EO exhibits potent antibacterial effects against *Escherichia coli*, *Staphylococcus aureus*, *Bacillus cereus*, *Bacillus subtilis*, *Bacillus laterosporus*, *Salmonella typhimurium*, and *Lactobacillus bulgaricus*, as well as antifungal effects against *Candida albicans* [163].

## 3. Safety of *Citrus* Oils

Generally speaking, *Citrus* EOs are non-toxic, non-mutagenic, and non-carcinogenic [8]. They are not hazardous in pregnancy and do not alter the maternal reproductive outcome [8,166]. Sweet orange, bitter orange, neroli, petitgrain, lemon, lime (both distilled and expressed), bergamot, and grapefruit oils have GRAS status [8]. However, there is a possible skin sensitization issue if old or oxidized oil is used. The distilled oils are not phototoxic, while the expressed oils carry a low to moderate risk of phototoxicity (Table 4) [167] due to the presence of furanocoumarins [168]. In case of applying expressed EOs to the skin in a dose higher than the maximum dermal use level, it is recommended to avoid exposure to sunlight for at least 12 h [8]. Neroli and yuzu oils are neither irritating nor sensitizing [167]. Expressed sweet orange oil was neither irritating nor sensitizing to 25 volunteers when tested at 8 and 100% [167], whereas it caused sensitivity to 0.13% of total dermatitis patients when tested at 2% [169]. Bitter orange EO was neither irritating nor sensitizing to 25 volunteers when tested at 10% [167], while it caused sensitivity to 1.5% of total dermatitis patients when tested at 2% [169]. Lemon oil was neither irritating nor sensitizing to volunteers when tested at 10% [167], and similar results were observed for distilled lime oil when tested at 15 and 100% [167]. No irritation or sensitization data were found for the expressed lime oil. The high citral content of lime EO causes potential toxic and myelotoxic effects [110]. Grapefruit oil was neither irritating nor sensitizing to volunteers when tested at 10 and 100% [167]. Mandarin EO was neither irritating nor sensitizing to 25 volunteers when tested at 5 and 8% [167]. The expressed bergamot oil was neither irritating nor sensitizing to 25 volunteers when tested at 10% [167]. It caused no irritation when tested at 2% on 1200 dermatitis patients, with only two (0.17%) patients showing sensitivity reaction [170], whereas when tested at 10% in 590 eczema patients, 0.5% of the patients had reactions [171]. Expressed bergamot oil caused severe phototoxic effects in hairless mice and pigs using simulated sunlight, and in humans using natural sunlight and may be photocarcinogenic [167]. When applied to mice, then irradiated with UV light, bergamot oil showed a carcinogenic action due to the presence of bergapten [172]. Chronic skin pigmentation (also known as berloque dermatitis, bergapten dermatitis, or photophytodermatitis) can also develop. Increased exposure to UV light can lead to serious burns [8]. In the absence of UV light, bergamot oil is not carcinogenic and even low concentration sunscreens can completely inhibit bergapten-enhanced phototumorigenesis [172]. No hazards found for the furanocoumarin-free (FCF) or rectified bergamot oil. The rectified oil was not sensitizing when tested at 30% on 25 volunteers [173].

To avoid oxidation of *d*-limonene, *Citrus* oils should be stored in a dark air-tight container and placed at 4 °C [8]. The use of old or oxidized oils should be avoided. To avoid any possible adverse skin reactions, it is recommended to dilute *Citrus* oils with a carrier oil before topical use [174]. Also, adding an antioxidant to preparations containing *Citrus* oils is recommended [8].

## 4. Bioactivity and Safety of Individual Key Components 

### 4.1. d-Limonene

*d*-Limonene has been shown to possess antioxidant, anti-inflammatory [12], and anticarcinogenic [8] effects. It is not acutely toxic, nephrotoxic, or carcinogenic, but the oxidized *d*-limonene may carry some toxicity. Unoxidized *d*-limonene is listed as an allergen by the EU, and moderately allergenic in Germany [8]. Unoxidized *d*-limonene was allergenic in 0.2% of dermatitis patients when tested at 2–3% [8]. No positive skin reactions were observed when testing the 98% pure *d*-limonene at 20% in dermatitis patients [175]. Undiluted *d*-limonene was moderately irritating to rabbits [167]. *d*-Limonene was irritating at concentrations of 70–80%, a weak irritant at 50%, and a non-irritant at concentrations of 20–30%. The acute dermal LD_50_ of *d*-limonene was >5 g/kg in rabbits, while the acute oral LD_50_ was >5 g/kg in rats [167].

### 4.2. γ-Terpinene

γ-Terpinene is an antioxidant [176]. It is neither irritating nor sensitizing [167]. It possesses minimal toxicity. Depending on concentration, it may be mutagenic or non-mutagenic [8]. The acute dermal LD_50_ of γ-terpinene was >5 g/kg in rabbits, while the acute oral LD_50_ was 3.65 g/kg in rats [167].

### 4.3. Linalool

Linalool is a sedative, an antidepressant, and an anticancer, antifungal, and pesticidal EO [177,178,179,180]. It is neither toxic nor irritable to skin. It presents an extremely low risk of skin sensitization [8]. No positive skin reactions were observed when testing the 97% pure linalool at 20%, or to oxidized linalool tested at 1% in dermatitis and eczema patients [175,181]. Linalool does not cause photo-irritation or photo-allergy because it does not absorb UV light in the range of 290–400 nm [182]. No fetal toxicity was observed [8]. No carcinogenic, mutagenic, or genotoxic activities were found [8]. The acute dermal LD_50_ was 5.61 g/kg in rabbits, while the acute oral LD_50_ was 2.79 g/kg in rats [183] and 2.2–3.92 g/kg in mice [184]. High doses of linalool cause ataxia and narcosis [185].

### 4.4. Linalyl Acetate

Linalyl acetate has narcotic effects [177]. It is non-toxic, and is very minimally skin reactive [8]. When tested at 5–20%, no skin reaction was observed [186]. Similar to linalool, linalyl acetate does not cause photo-irritation or photo-allergy because it does not absorb UV light in the range of 290–400 nm [182]. It has no carcinogenic activity [8]. The acute dermal LD_50_ was higher than 5 g/kg in rabbits, while the acute oral LD_50_ was 14.5 g/kg in rats and 13.5 g/kg in mice [184].

### 4.5. α-Terpineol

α-Terpineol has anticarcinogenic activity [187]. It is a non-irritant at 1–15%, and non-phototoxic [188]. It is not mutagenic or genotoxic. The acute dermal LD_50_ of the mixed isomer terpineol was >3 g/kg in rabbits, while the acute oral LD_50_ was 4.3 g/kg in rats [167].

### 4.6. Geranyl Acetate

Geranyl acetate has anti-inflammatory [189], antifungal [189], and antimicrobial properties [190]. It is a very weak skin sensitizer [167]. It is neither toxic nor carcinogenic [8]. It was not mutagenic in the Ames test [191], and had no genotoxic effect [192]. The acute oral LD_50_ of geranyl acetate is 6.33 g/kg in rats [183].

### 4.7. Terpinolene

Terpinolene is an antioxidant [193]. It is neither irritating nor sensitizing at 20% [167]. Limited data suggests minimal toxicity. The acute oral LD_50_ was 4.4 mL/kg in rats and mice [167]. Thresholds of terpinolene skin sensitization are not known.

### 4.8. β-Pinene

β-Pinene showed antiproliferative and cytotoxic effects [19,194]. It is not mutagenic or genotoxic [8]. It is generally a non-irritant and non-sensitizing. Undiluted β-pinene was moderately irritating to rabbits [8]. β-pinene was irritating at concentrations of 70–80%, a weak irritant at 50%, and a non-irritant at concentrations of 25–30% to dermatitis patients [195]. β-Pinene was classified as a category B substance in Germany, meaning it is considered moderately allergenic [196]. The acute dermal LD_50_ of β-pinene was >5 g/kg in rabbits, subcutaneous LD_50_ was 1.42 g/kg in mice, and the acute oral LD_50_ was >5 g/kg in rats [167].

## 5. Conclusions

*Citrus* essential oils are well known for their flavor and fragrance properties, as well as numerous aromatherapeutic and medicinal applications. With the exception of some phototoxicity of expressed oils, they are generally safe to use with negligible toxicity to humans. These readily available essential oils will undoubtedly continue to play important roles in the food and beverage industries, as well as for medicinal, cosmetic, and “green” pest-control uses.

## Figures and Tables

**Table 1:** Major volatile components in essential oils of different *Citrus* spp.

*Citrus* EO		Sweet Orange [8,21]	Bitter Orange [8]	Neroli (Egyptian) [8]	Petitgrain [8]	Mandarin [8]	Lemon (D) [8]	Lemon (Ex) [8]	Lime (D) [8,22,23]	Lime (Ex) [8,22]	Bergamot (FCF) [8,24]	Bergamot (Ex) [8,24,25]	Grapefruit [8,26]	Yuzu [8,27]
Plant Part		Fruit Peel	Fruit Peel	Flower	Leaf	Fruit Peel	Fruit Peel	Fruit Peel	Fruit Peel	Fruit Peel	Fruit Peel	Fruit Peel	Fruit Peel	Fruit Peel
**Essential oil Composition**	*d*-Limonene	83.9–95.9%	89.7–94.7%	6.0–10.2%	0.4–8.0%	65.3–74.2%	64.0–70.5%	56.6–76.0%	40.4–49.4%	48.2%	28.0–45.0%	27.4–52.0%	84.8–95.4%	63.1%
	Linalool	0–5.6%	0.1–2.0%	43.7–54.3%	12.3–24.2%						4.0–20.0%	1.7–20.6%		2–8%
	Linalyl acetate			3.5–8.6%	51.0–71.0%						18.0–28.0%	17.1–40.4%		
	β-Pinene			3.5–5.3%	0.3–2.7%	1.4–2.1%	8.2–14.0%	6.0–17.0%	2.0–2.9%	21.1%	4.0–11.0%	4.4–11.0%		1.1%
	γ-Terpinene					16.4–22.7%	8.4–10.7%	3.0–13.3%	9.5–10.7%	8.1%	3.0–12.0%	5.0–11.4%		12.5%
	α-Pinene	0.6–1.0%				2.0–2.7%	1.1–2.1%	1.3–4.4%	1.2–2.1%	2.5%	1.0–1.8%	0.7–2.2%	0.2–1.6%	2.7%
	β-Myrcene	1.3–3.3%	1.6–2.4%	1.4–2.1%	0–2.0%	1.5–1.8%	1.4–1.6%	tr–2.2%	1.3–2.1%	1.3%		0.6–1.8%	1.4–3.6%	3.2%
	α-Terpineol			3.9–5.8%	2.1–5.2%			0.1–8.0%	5.4–12.7%					
	(*E*)-β-Ocimene			4.6–5.8%	0.2–2.2%									
	Sabinene	0.2–1.0%		0.4–1.6%			0.8–1.7%	0.5–2.4%		3.1%			0.4–1.0%	
	Neral	0–1.3%					0.5–1.5%	0.4–2.0%		1.4%				
	Geranial	0–1.8%					0.7–2.2%	0.5–4.3%		2.4%				
	Bicyclogermacrene													2.0%
	(*E*)-β-Farnesene													1.3%
	Geranyl acetate			3.4–4.1%	1.9–3.4%									
	Terpinolene					0.7–1.0%			8.1–8.7%					
	(*E*)-Nerolidol			1.3–4.0%										
	Geraniol			2.8–3.6%	1.4–2.3%									
	Nerol			1.1–1.3%	0.4–1.1%									
	*p*-Cymene					0.1–1.4%		tr–2.3%	1.6–2.5%					
	(*E*,*E*)-Farnesol			1.6–3.2%										
	(*E*,*Z*)-Farnesol													
	Neryl acetate			1.7–2.1%	0–2.6%			0.1–1.5%				0.1–1.2%		
	Terpinen-4-ol							tr–1.9%	0.7–1.9%					
	(*Z*)-β-Ocimene			0.7–1.0%										
	α-Thujene					0.7–1.0%								
	1,4-Cineole								2.0–3.0%					
	Terpinen-1-ol								1.0–2.3%					
	(*Z*)-β-Terpineol								0.5–2.2%					
	α-Terpinene								tr–2.1%					
	β-Bisabolene								1.6–1.8%	1.8%				
	α-Fenchol								0.6–1.4%					
	Borneol								0.5–1.4%					
	Camphene								0.5–1.3%					
	γ-Terpineol								0.8–1.6%					
	(*E*)-α-Bergamotene									1.1%				
	β-Caryophyllene									1.0%				
	(2*E*,6*E*)-α-Farnesene									1.0%				
	β-Phellandrene													5.4%
	Nootkatone												0.1–0.8%	

D = Distilled; Ex = Expressed; FCF = Furanocoumarin-Free.

**Table 2:** Non-volatile components of some expressed *Citrus* oils.

Non-Volatile Components	Bitter Orange [8,26]	Lemon [8]	Lime [8,26]	Grapefruit [8,26]	Bergamot [8,24,25]	Bergamot (FCF) [8,24]	Mandarin [8]
Bergamottin	-	0.16–0.54%	1.7–3.0%	<0.11%	0.68–2.75%	0–1.625%	0–0.001%
Bergapten	0.035–0.073%	0.0001–0.035%	0.17–0.33%	0.012–0.19%	0.11–0.33%	0–0.0091%	0–0.0003%
Oxypeucedanin	-	0.09–0.82%	0.02–0.3%	-	-	-	-
5-Geranloxy-7-methoxycoumarin	-	0.18–0.28%	1.7–3.2%	-	0.08–0.68%	0–0.19%	-
Citropten	-	0.05–0.17%	0.4–2.2%	-	0.01–0.35%	0–0.0052%	-
Byakangelicol	-	0.006–0.16%	-	-	-	-	-
8-Geranyloxypsoralen	-	0.01–0.045%	0.10–0.14%	-	-	-	-
Isopimpinellin	-	0–0.011%	0.1–1.3%	-	-	-	-
5-Geranoxy-8-methoxypsoralen	-	-	0.2–0.9%	-	-	-	-
Epoxybergamottin	0.082%	-	-	0.1126%	-	-	-
Psoralen	0.007%	-	-	-	0–0.0026%	-	-
Bergaptol	-	-	-	-	0–0.19%	-	-

FCF = Furanocoumarin-Free.

**Table 3:** Biological activities of different *Citrus* essential oils.

Essential Oil	Biological Activity	Ref.
Sweet orange	Anticarcinogenic	[28,29]
	Relaxant	[30]
	Anxiolytic	[31,32,33]
	Pain relief	[34]
	Hepatocarcinogenesis suppressant	[35]
	Anti-tumor	[36]
	Antioxidant	[37]
	Food preservative	[38]
	Acne treatment (with sweet basil oil)	[39]
	Antibacterial	[40,41,42,43]
	Antifungal	[10,44,45]
	Anti-aflatoxigenic (at 500 ppm)	[44]
	Larvicidal	[46,47]
	Insecticidal	[48,49,50]
	Anthelmintic	[51]
	Growth promoter (in Tilapia)	[52]
Bitter orange	Mild sedative, hypnotic, soothing, calming, and motor relaxant	[53]
	Sleep inducer	[54]
	Anxiolytic and antidepressant	[53,55,56,57,58]
	Pain relief	[34,59]
	Antiseizure and anticonvulsant agent	[54]
	Anti-spasmodic and sexual desire enhancer	[59]
	Gastroprotective and ulcer healing	[60]
	Digestive disorders treatment	[53]
	Hepatocarcinogenesis suppressant	[35]
	Antioxidant	[53,61]
	Nephroprotective	[62]
	Antibacterial	[53,63,64,65]
	Pimple and acne treatment	[53]
	Antifungal	[15,53,66]
	Fumigant and anti-cholinesterase	[67]
	Larvicidal	[46]
Neroli	Sedative, soothing, calming, and motor relaxant	[55,68]
	Anxiolytic and antidepressant	[53,57,69,70]
	Antiseizure and anticonvulsant	[71,72]
	Central and peripheral antinociceptive effects	[73]
	Anti-inflammatory	[73]
	Menopausal symptoms relief	[74]
	Premenstrual syndrome (PMS) relief	[75]
	Sexual desire enhancer	[59]
	Endothelium- and smooth muscle-dependent vasodilator	[76]
	Hypotensive	[77]
	Antioxidant	[20,78]
	Anti-amnesic	[72]
	Antibacterial	[53,79]
	Antifungal	[20,53,75,79]
Orange petitgrain	Antioxidant	[78,80]
	Antibacterial	[81]
	Antifungal	[81]
Mandarin	Anti-proliferative	[82]
	Chemoprotective	[82]
	Antioxidant	[83]
	Antibacterial	[83,84]
	Antifungal	[84,85,86,87]
Lemon	Stress relief	[88,89]
	Cytotoxic	[28,90]
	Chemoprotective	[91]
	Anti-obesity	[92]
	Antioxidant	[93]
	Neuroprotective	[94,95]
	Anti-anxiety	[96]
	Creativity and mood enhancer	[97]
	Analgesic	[98]
	Relief of nausea and vomiting of pregnancy	[99]
	Anti-spasmodic	[89]
	Attention level, concentration, cognitive performance, mood, and memory enhancer	[89,100]
	Skin penetration enhancer	[101]
	Antibacterial	[102,103]
	Antifungal	[10]
	Insect repellent	[104]
	Miticidal	[105]
Lime	Anti-obesity	[106]
	Spasmolytic agent	[107,108]
	Selective acetylcholinesterase and buytrylcholinesterase inhibitor	[109]
	Antioxidant	[109]
	Anti-inflammatory	[110]
	Flavoring agent	[111,112]
	Antibacterial	[111,113]
	Antifungal	[111,113]
	Insecticidal	[114]
	Phytotoxic	[113]
Grapefruit	Anti-obesity	[92,115,116,117]
	Cravings and hunger reducer (mixed with patchouli oil)	[116]
	Body cleansing promoter	[116]
	Cytotoxic	[28,90]
	Antibacterial	[118,119]
	Antifungal	[118,119,120]
	Larvicidal	[121,122,123,124]
Bergamot	Melanogenic component in suntan preparations	[125,126]
	Pain relief	[127,128,129]
	Peripheral antinociceptive	[129,130]
	Antiallodynic	[127,131]
	Wound healing	[132]
	Cytotoxic	[125,133,134,135]
	Anti-tumor	[136]
	Neuroprotective	[137,138]
	Sedative, calming, and soothing	[139]
	Anxiolytic	[139,140]
	Mood enhancer	[141]
	Antioxidant	[109]
	Antibacterial	[142,143,144]
	Antifungal	[142,143,145]
	Anti-dermatophyte	[146,147]
	Antimycoplasmal	[148]
Yuzu	Anti-carcinogenic	[149]
	Anti-inflammatory	[150]
	Anti-anxiety	[151]
	Mood disturbance, tension-anxiety, anger-hostility, and fatigue reducer	[152,153]
	Mind and body health promoter	[152]
	Odor suppressant	[154]
	Anti-cancer	[155]
	Hypocholesterolemic	[156]
	Anti-diabetic	[157]
	Anti-obesity	[158]
	Platelet aggregation inhibitor	[159]
	Heart failure treatment	[160]
Kumquat	Antiproliferative	[161]
	Antioxidant	[161,162]
	Antibacterial	[163]
	Antifungal	[163]

**Table 4:** Phototoxicity risk, irritation of the undiluted oil, acute dermal LD_50_ in rabbits, acute oral LD_50_ in rats, and maximum dermal use level for different essential oils from *Citrus* species.

Acute Toxicity	Phototoxicity Risk [167]	Irritation of Undiluted Oil [8]	Acute Dermal LD_50_ in Rabbits (g/kg) [167]	Acute Oral LD_50_ in Rats (g/kg) [167]	Maximum Dermal Use Level [8]
Sweet orange EO	Low risk	Moderately irritating to rabbits but not irritating to mice	>5	>5	-
Bitter orange EO	low risk	Moderately irritating to rabbits	>10	>5	1.25%
Neroli EO	Not phototoxic	Not irritating	>5	4.55	-
Petitgrain EO	Not phototoxic	Slightly irritating to rabbits, but not irritating to mice or pigs	<2	>5	-
Lemon EO (distilled)	Not phototoxic	Moderately irritating to rabbits and slightly irritating to mice	>5	>5	20%
Lemon EO (expressed)	Low risk	Not irritating	>5	>5	2%
Lime EO (distilled)	Not phototoxic	Slightly irritating to rabbits	>5	>5	-
Lime EO (expressed)	moderate risk	No data available	>5	>5	0.7%
Grapefruit EO	Low risk	Slightly irritating to rabbits, but not irritating to mice or pigs	>5	>5	4%
Bergamot EO (FCF)	Not phototoxic	Mildly irritating to rabbits	>20	>10	0.4%
Bergamot EO (expressed)	Moderate risk	Moderately irritating to rabbits	-	-	-
Yuzu EO	Not phototoxic	Not irritating	-	-	-
Mandarin	Not phototoxic	Moderately irritating (produces slight edema and erythema) to rabbits, mice, and pigs	>5	>5	30%

## Abbreviations

5-HT	serotonin
ACC	anterior cingulate cortex
AMPK	AMP-activated protein kinase
BLM	bleomycin
COX-2	cyclooxygenase-2
CTGF	connective tissue growth factor
DA	dopamine
DBP	Dibenzo-[α]-pyrene
DENA	*N*-nitrosodiethylamine
DPPH	2,2-diphenyl-1-picrylhydrazyl
EO	essential oil
EPM	elevated plus-maze
ERK	extracellular signal-regulated protein kinase
FCF	furanocoumarin-free
GABA	gamma-aminobutyric acid
GRAS	generally recognized as safe
GSH	glutathione
HELFs	human embryonic lung fibroblasts
HL-60	human leukemia cells
HPA	hypothalamic-pituitary-adrenal
IC_50_	median inhibitory concentration
IR	insulin resistance
LD_50_	median lethal dose
LDL	low-density lipoprotein
LNCaP	human prostate acedocarcinoma cells
MI	myocardial infarction
NDMA	*N*-nitrosodimethylamine
NNK	4-(methylnitrosoamine)-1-(3-pyridyl)-1-butanone
NVP	nausea and vomiting of pregnancy
phospho-Akt	phosphorylated protein kinase B
phospho-GSK-3β	phosphorylated glycogen synthase kinase 3 beta
PMS	premenstrual syndrome
PPAR-γ	peroxisome proliferator-activated receptor gamma
ppm	parts per million
ROS	reactive oxygen species
RyR	ryanodine receptor
SH-SY5Y	human neuroblasoma cells
WAT	white adipose tissue
